# High Incidence and Impact of Suspected Exocrine Pancreatic Insufficiency in Patients Post‐Hematopoietic Stem Cell Transplantation: A Single‐Center Prospective Observational Study

**DOI:** 10.1002/ueg2.12769

**Published:** 2025-02-15

**Authors:** Michael Launspach, Aurika Mindermann, Joachim Schulz, Lina Alasfar, Sandra Cyrull, Felix Zirngibl, Lena Oevermann, Annette Künkele, Hedwig E. Deubzer, Horst von Bernuth, Axel Pruß, Peter Lang, Philip Bufler, Angelika Eggert, Arend von Stackelberg, Johannes H. Schulte

**Affiliations:** ^1^ Department of Pediatric Oncology and Hematology Charité ‐ Universitätsmedizin Berlin Berlin Germany; ^2^ Berlin Institute of Health at Charité ‐ Universitätsmedizin Berlin Berlin Germany; ^3^ The German Cancer Consortium (DKTK) Partner Site Berlin Berlin Germany; ^4^ The German Cancer Research Center (DKFZ) Heidelberg Germany; ^5^ Department of Internal Medicine V University Hospital Heidelberg Im Neuenheimer Feld 672 Heidelberg Germany; ^6^ Experimental and Clinical Research Center Max Delbrück Center for Molecular Medicine and Charité ‐ Universitätsmedizin Berlin Berlin Germany; ^7^ Department of Pediatric Respiratory Medicine Immunology and Critical Care Medicine Charité ‐ Universitätsmedizin Berlin Berlin Germany; ^8^ Department of Immunology Labor Berlin ‐ Charité Vivantes GmbH Berlin Germany; ^9^ Berlin‐Brandenburg Center for Regenerative Therapies (BCRT) Charité ‐ Universitätsmedizin Berlin Berlin Germany; ^10^ Institute of Transfusion Medicine Charité ‐ Universitätsmedizin Berlin Berlin Germany; ^11^ Department of Hematology/Oncology and General Pediatrics Children’s University Hospital University of Tuebingen Tuebingen Germany; ^12^ Department of Pediatric Gastroenterology, Nephrology, and Metabolic Diseases Charité ‐ Universitätsmedizin Berlin Berlin Germany

**Keywords:** adenovirus infection, bone marrow transplantation, exocrine pancreas function, fecal elastase, immunosuppression, pancreas elastase, pancreas imaging, pancreas ultrasound, pancreatitis, stem cell transplantation

## Abstract

Exocrine pancreatic insufficiency (EPI) is suspected but remains understudied in immunosuppressed conditions such as post‐hematopoietic stem cell transplantation (HSCT). This prospective observational study aimed to investigate the incidence, impact, and risk factors of EPI in a cohort of 83 pediatric and young adult patients who underwent allogeneic HSCT at Charité ‐ Universitätsmedizin Berlin between 2020 and 2023. Fecal pancreatic elastase (PE) measurements and transabdominal ultrasound were utilized to evaluate pancreatic function over a one‐year period. Secondary analysis explored the association of EPI with clinical complications and included a multivariable regression analysis of potential risk factors. Low PE levels significantly correlated with pathological pancreatic imaging findings, independent of concurrent diarrhea. EPI was suspected in 45% (32/71) of patients (95%CI: [34.1%, 56.6%]), with 29% (13/45) (95%CI: [17.7%, 43.4%]) showing signs of prolonged EPI (pEPI) lasting at least 8 weeks. After excluding cases with confounding factors such as missing enteral nutrition and diarrhea, the cumulative incidence of prolonged EPI was 20% (8/41) (95%CI: 10.2%–34.0%) in the overall cohort. EPI was associated with weight loss, prolonged dependence on parenteral nutrition, and extended hospitalizations. Adenovirus (ADV) infection emerged as a significant risk factor for EPI (hazard ratio 3.22 [95%CI:1.26–8.2], *p* = 0.014), along with additional factors such as higher BMI pre‐HSCT, pre‐existing pancreatic damage and early post‐HSCT fasting. The persistence of pancreatic atrophy and EPI beyond two years post‐HSCT in individual cases suggests a potential for permanent pancreatic damage. This study underscores that EPI is a common complication following HSCT, with ADV infection being an important risk factor. The findings support routine PE measurements and early initiation of pancreatic enzyme replacement therapy (PERT), alongside aggressive treatment of ADV infections. Further research is necessary to evaluate the effects of PERT in this population and to explore the applicability of these findings in other immunosuppressed groups.

## Graphical abstract

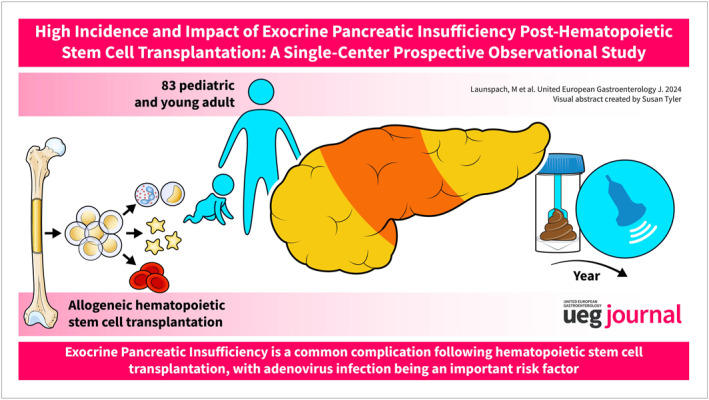

1

**Summary**

- Summarize the established knowledge on this subject
  ◦ Exocrine pancreatic insufficiency (EPI) is an underdiagnosed condition characterized by insufficient pancreatic enzyme delivery, causing nutrient maldigestion and deficiencies, with diagnosis primarily relying on fecal elastase testing and clinical symptom assessment.
  ◦ EPI is suspected to occur in immunosuppressed patients, such as those undergoing hematopoietic stem cell transplantation (HSCT), but has been underexplored in terms of its incidence, risk factors, and clinical impact.
- What are the significant and/or new findings of this study?
  ◦ This study reveals a high incidence of prolonged EPI post‐HSCT, and identifies adenovirus (ADV) infection as a significant risk factor, along with other factors, highlighting the potential for prolonged or permanent pancreatic damage and the need for routine monitoring and early intervention in immunosuppressed patients.
  ◦ A significant correlation between pancreatic elastase levels and transabdominal ultrasound findings highlights the potential role of cross‐sectional imaging in aiding EPI diagnosis post‐HSCT, warranting further investigation.

**Abbreviations**
a/c GvHD: acute/chronic graft versus host disease
ADV: adenovirus
BMI: body mass index
CMV: cytomegalovirus
d: day
EBV: epstein‐barr virus
GI: gastrointestinal
HHV6: human herpesvirus 6
HIV: human immunodeficiency virus
HR: hazard ratio
HSCT: hematopoietic stem cell transplantation
OR: odds ratio
PE: pancreatic elastase
PERT: pancreatic enzyme replacement therapy
RR: relative risk
s.(p)EPI: both suspected exocrine pancreatic insufficiency and suspected prolonged exocrine pancreatic insufficiency analysis groups
s.EPI: suspected exocrine pancreatic insufficiency
s.pEPI: suspected prolonged exocrine pancreatic insufficiency
TBI: total body irradiation
w: week
95%CI: 95% confidence interval

## 2 Introduction

Allogeneic hematopoietic stem cell transplantation (HSCT) conditioning regimens and complications such as graft‐versus‐host disease (GvHD) or infections (e.g., Adenovirus) frequently result in severe organ dysfunction [1]. Although many of these complications manifest with diarrhea, abdominal pain, malabsorption and growth failure or weight loss, frequently no specific cause for these symptoms is found [2]. At the same time, these symptoms are associated with exocrine pancreatic insufficiency (EPI) [3]. EPI, defined as a reduction in exocrine pancreatic secretion or pancreatic enzyme activity below levels required for normal nutrient digestion, results in maldigestion and can be treated by pancreatic enzyme replacement therapy (PERT) [4, 5, 6]. The most well‐known causes for EPI are chronic pancreatitis and cystic fibrosis, but other causes like inflammatory bowel diseases have been identified as well and EPI remains underdiagnosed [7]. Pancreatitis is a known complication of HSCT, with some data indicating that adenoviral infections during immunosuppression can cause chronic pancreatitis [8, 9, 10]. Despite these communalities, the incidence and risk factors of EPI after allogeneic HSCT remain unclear. Over the last two decades, six HSCT patients with severe EPI have been reported [11, 12, 13]. While standard diagnostic criteria are missing, an indirect and noninvasive way to assess EPI is the enzyme immunoassay of fecal pancreatic elastase (PE). It is a proteolytic enzyme secreted by the exocrine pancreas, which is excreted in unchanged concentrations in feces. Good correlations between PE and pancreatic enzyme output have been shown and PE testing is superior to alternative tests [14, 15, 16]. It is recommended for investigating chronic diarrhea and PE levels < 200 µg/g stool indicate EPI, with high sensitivity and specificity for values < 100 µg/g [5, 17, 18, 19]. Transabdominal sonography can detect signs of pancreatitis such as an echo‐dense, lobulated, or irregular organ contour and can identify primary causes of EPI [20, 21].

To elucidate the incidence and impact of EPI post‐HSCT, we conducted a prospective study involving 83 pediatric and young adult patients, utilizing PE measurement and transabdominal ultrasound.

## 3 Methods

### 3.1 Study Design and Patients

**FIGURE 1:**
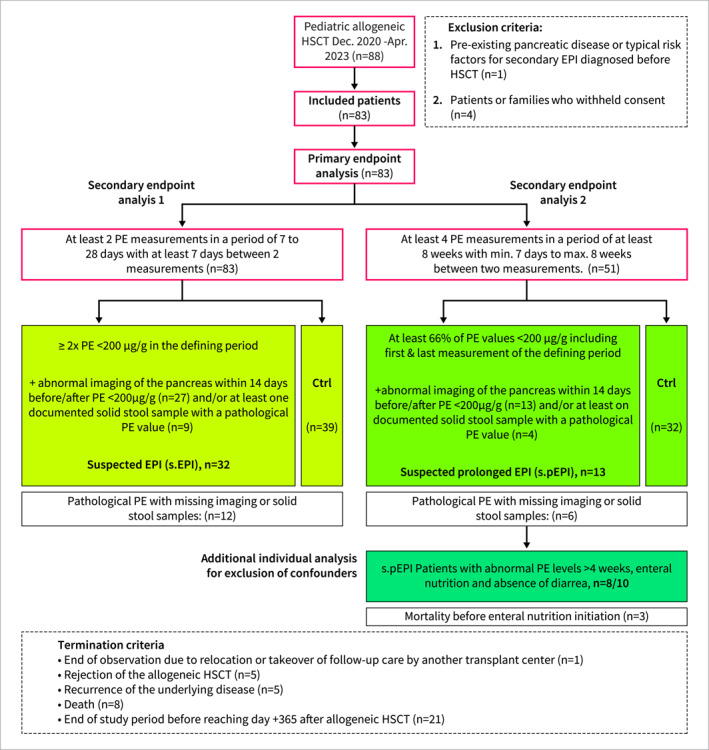
Study design and analysis overview. Sequential analysis flow including exclusion and termination criteria. Pancreatic diseases or typical risk factors for EPI considered for the exclusion criteria were: alcoholism, chronic pancreatitis, congenital absence or malformation, pancreatic tumors or atrophy, pancreatic resection or upper GI surgery, celiac disease, diabetes mellitus, inflammatory bowel disease, HIV infection, genetic causes like cystic fibrosis, zollinger‐ellison‐, shwachman‐diamond‐, johanson‐blizzard syndrome or hemachromatosis. Apr.: april, Ctrl: control group, Dec.: december, HSCT: hematopoietic stem cell transplantation, n: number of subjects, PE: pancreas elastase, s.(p)EPI: suspected (prolonged) exocrine pancreas insufficiency.

This prospective observational cohort study was conducted at the stem cell transplantation center of the Department of Pediatric Oncology and Hematology at Charité—Universitätsmedizin Berlin, Germany. All patients received myeloablative conditioning and allogeneic HSCT between December 14, 2020, and April 30, 2023 with follow‐up extending to d365 post‐HSCT or until June 15, 2023. The study, approved by the local ethics committee in accordance with the Declaration of Helsinki (Approval Date: 06.05.2021; File Number: EA2/098/21), included patients aged 2 months to 22 years who underwent allogeneic HSCT without pre‐existing pancreatic disease or common secondary EPI risk factors (Figure S1, Table S1). Exclusion and termination criteria are provided in Figure 1. Written informed consent was obtained from all participants and caretakers. The primary endpoints were: (1) longitudinal changes in stool PE levels measured at fixed time points (before HSCT (week (w) −2), during conditioning (w‐1) and w1, w2, w3, w4, day (d) 30, d60, d100, d180 and d365 after HSCT), (2) correlation of PE values with stool consistency, and (3) association of pancreatic imaging findings with PE levels. Secondary endpoints included: (1) evaluation of group allocation criteria for suspected EPI (s.EPI) and suspected prolonged EPI (s.EPI) and (2) explorative comparisons of s.(p)EPI patients with control groups for clinical parameters, outcomes and secondary complications, supplemented by multivariable risk factor analysis. Group allocation criteria are described in Figure 1. Pancreatic enzyme replacement therapy (PERT) was not part of the study and if started it was done so by unaffiliated physicians. PERT was not considered a dropout criterion as it does not impact fecal PE levels and the primary study endpoints.

### 3.2 Data Collection

PE measurements were performed using the DiaSorin LIAISON Elastase‐1 Assay Kit on a LIAISON XL Analyzer. Watery samples were lyophilized to reduce water content and false‐positive risk [22, 23]. A single senior investigator (DEGUM course leader–level II, pediatric sonography) conducted standardized pancreatic ultrasound imaging for all patients in a blinded manner during routine abdominal ultrasounds (GE Logiq E9 device, 9L or C1‐6 probes). The pancreas was imaged transversely in the upper abdomen, with patients distending their abdomen for optimal assessment. Pancreatic morphology was described in terms of size, texture and echogenicity.

### 3.3 Statistical Analysis

Statistical analyses were conducted using GraphPad PRISM 8 & 9 (GraphPad Software, San Diego, United States of America) and *R* version 4.3.3 (R Foundation). Kaplan‐Meier statistics and a multivariable Cox regression risk factor analysis model were used for time‐to‐event analyses. Comparative analyses were performed using non‐parametric and parametric tests as appropriate, with a significance level of *p* < 0.05 considered statistically significant. Detailed descriptions of the statistical methods are provided in the supplementary material and figure legends.

## 4 Results

### 4.1 High Incidence of EPI Post‐HSCT

**FIGURE 3:**
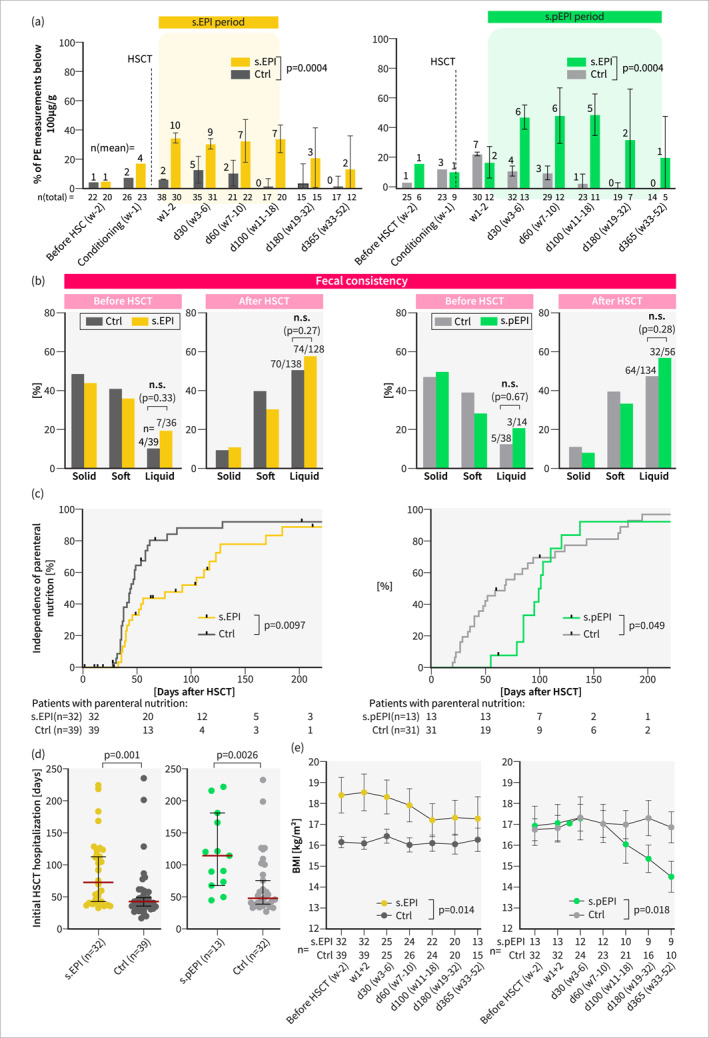
s.(p)EPI group allocation is associated with increased secondary complications. (a) Proportions of PE measurements below 100 μg/g in s.(p)EPI‐ and control groups. (b) Comparison of fecal consistency distribution (solid, soft, or liquid) before and after HSCT between s.(p)EPI‐ and control groups. (c) Inverse Kaplan‐Meyer curves depicting the rate of s.(p)EPI‐ and control patients independent of parenteral nutrition over time. (d) Comparison of initial hospitalization duration between s.(p)EPI‐ and control groups. (e) Mean BMI development over time, calculated for s.(p)EPI‐ and control groups.(p)EPI: suspected (prolonged) exocrine pancreas insufficiency, d/w: days/weeks after HSCT, Ctrl: control group, HSCT: hematopoietic stem cell transplantation, PE: pancreas elastase, n: sample size. Statistical analysis: (a, e) Mixed‐model test, (b) Fisher’s exact test, (c): Gehan‐Breslow‐Wilcoxon test, (d) Mann‐Whitney Test; n.s.: not significant.

Analysis of PE levels across all patients revealed a significant drop early post‐HSCT, reaching its nadir on day 30. Correspondingly, the proportion of patients with mean PE levels < 200 µg/g significantly increased post‐HSCT 42% (32/77) on d30 versus 8% (4/50) pre‐HSCT; 95% confidence interval (95%CI) of difference: [+20.2%, +46.9%] Here we observed a significant increase already during conditioning (w‐1) (Figure 2a and b). As expected, we observed lower PE levels in liquid stool samples (Figure S2E and F). To address potential confounding by diarrhea, we performed a separate analysis restricted to solid samples (69 samples from 42 patients), that revealed a significantly higher percentage of patients with pathological PE levels in solid samples during conditioning (w‐1, 21% (5/24) and early post‐HSCT (w1‐4, 21% (9/42)) compared to healthy children from a historic control cohort (1.3% (3/227)) (95%CI: [+3%, +39%]). Pre‐HSCT versus conditioning or Post‐HSCT comparison showed an increase but no statistical significance (Figure 2c) [24]. Strikingly, patients with at least two stool samples showing PE levels < 200 µg/g were significantly more likely to exhibit abnormal pancreas imaging findings post‐HSCT characterized by increased echogenicity and irregular organ texture, irrespective of fecal consistency and time point post‐HSCT (OR 3.9) (Figure 2d–f). For subsequent secondary analysis, we used pre‐tested group allocation criteria that demonstrated the highest discriminatory power (Figure 1, Figure S2). Analysis 1 resulted in 45% (32/71), 95%CI: [34.1%, 56.6%] suspected EPI (s.EPI) patients and analysis 2 in 29% (13/45), 95%CI: [17.7%, 43.4%]) patients with suspected prolonged (s.pEPI) (Figure 1). All s.pEPI cases had been allocated to the s.EPI group, resulting in a 57% (95%CI: [36.3%, 76.8%]) risk for s.EPI patients to develop s.pEPI. To validate our approach, we assessed PE levels and stool consistency across all subgroups. s.(p)EPI patients showed a highly significant proportion of PE levels below 100 µg/g, indicative of severe insufficiency (Figure 3a). All s.(p)EPI and control patients displayed similar proportions of soft or liquid stool samples throughout the study period and we found significantly lower PE levels both in solid and in liquid solid stool samples of s.EPI patients (Figure 3b, Figure S4). As we observed a strong correlation between food intake and PE levels (Figure S2G) and since missing enteral nutrition can lower baseline exocrine secretion and result in abnormal pancreatic elastase (PE) levels despite normal pancreatic function, we performed an individual analysis of patients in the s.pEPI group. Findings revealed that 80% (8/10) exhibited abnormally low PE levels for over four weeks, despite receiving full enteral nutrition and being free from diarrhea. Three patients were excluded due to early mortality before enteral nutrition initiation. This indicates a high probability of clinically relevant EPI in these cases, leading to a cumulative incidence of clinically relevant prolonged EPI in the overall cohort of 20% (8/41; 95%CI: 10.2%–34.0%).

FIGURE 2Analyses of PE levels and pancreas imaging suggest a high incidence of EPI post‐HSCT. Mean PE level per patient and time period. Statistical analysis: (a) Kruskal‐Wallis test and Dunn post hoc test comparing different time‐points against before HSCT, (B‐D,F): Fisher's exact test; n.s.: not significant. (b) Patients with mean PE levels < 200 μg/g stool per time period. (c) Patients with PE < 200 μg/g in solid stool samples at different time points compared to a historic control cohort. (d) Rate of patients with sonographic pancreas findings, regardless of imaging time point, grouped by PE level findings. (e) Representative sonographic images of the pancreas of an unaffected control compared to a s.pEPI patient (female, age 8–14). Abnormal pancreatic findings included increased echogenicity, irregular texture, and signs of atrophy (reduced size). (f) Comparison of the occurrence rate of pathological and normal imaging findings (Image +/−) between s.(p)EPI‐ and control groups. For s.(p)EPI groups only imaging was considered that was conducted after s.(p)EPI onset or latest 14 days after the last abnormal PE level. s.(p)EPI: suspected (prolonged) exocrine pancreas insufficiency, Ctrl: control group, d/w: day/week after HSCT, HSCT: hematopoietic stem cell transplantation, PE: pancreas elastase, n: sample size.

**Figure:**
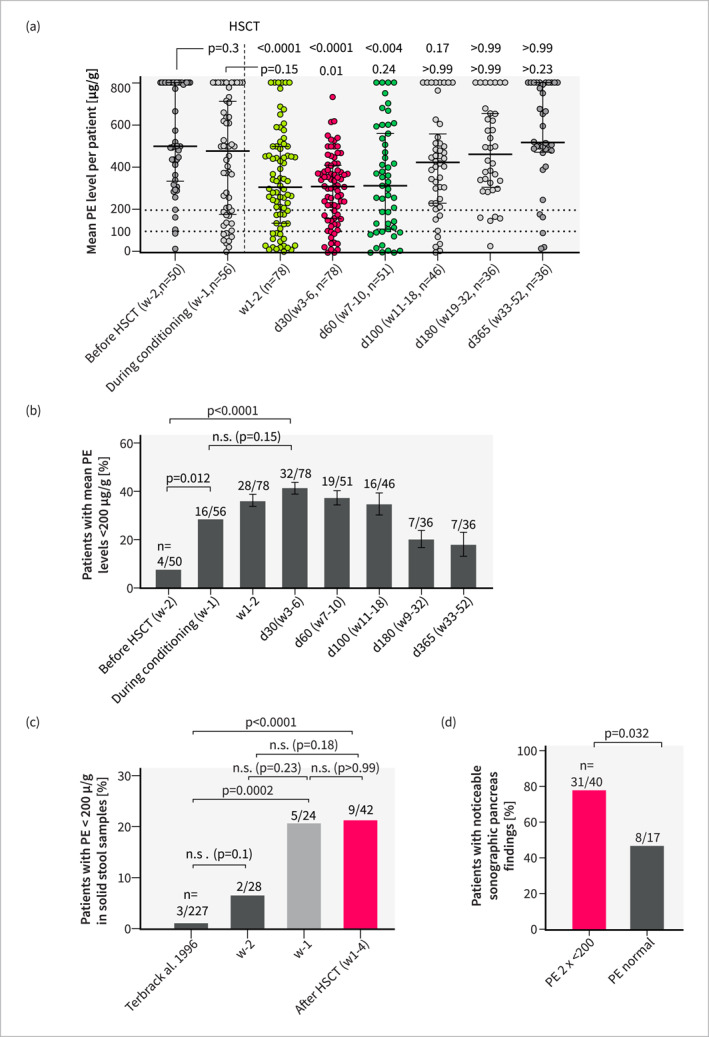

**Figure:**
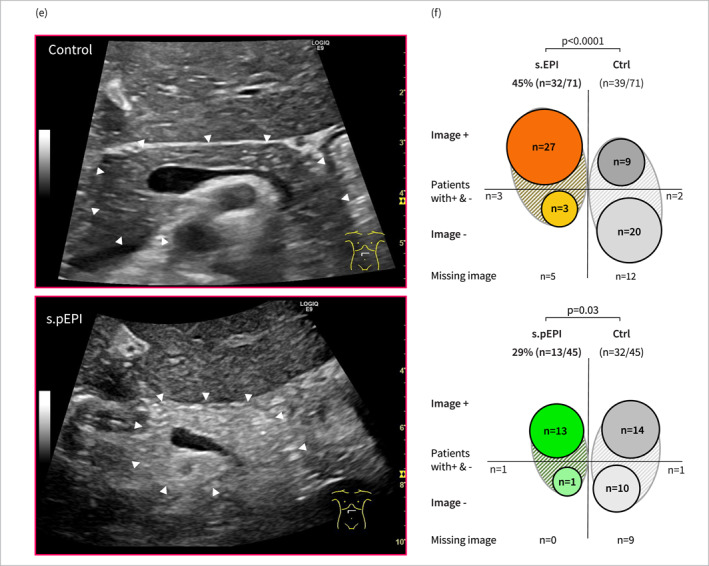

### 4.2 EPI Group Allocation is Associated With Higher Secondary Complication Rates

**FIGURE 4:**
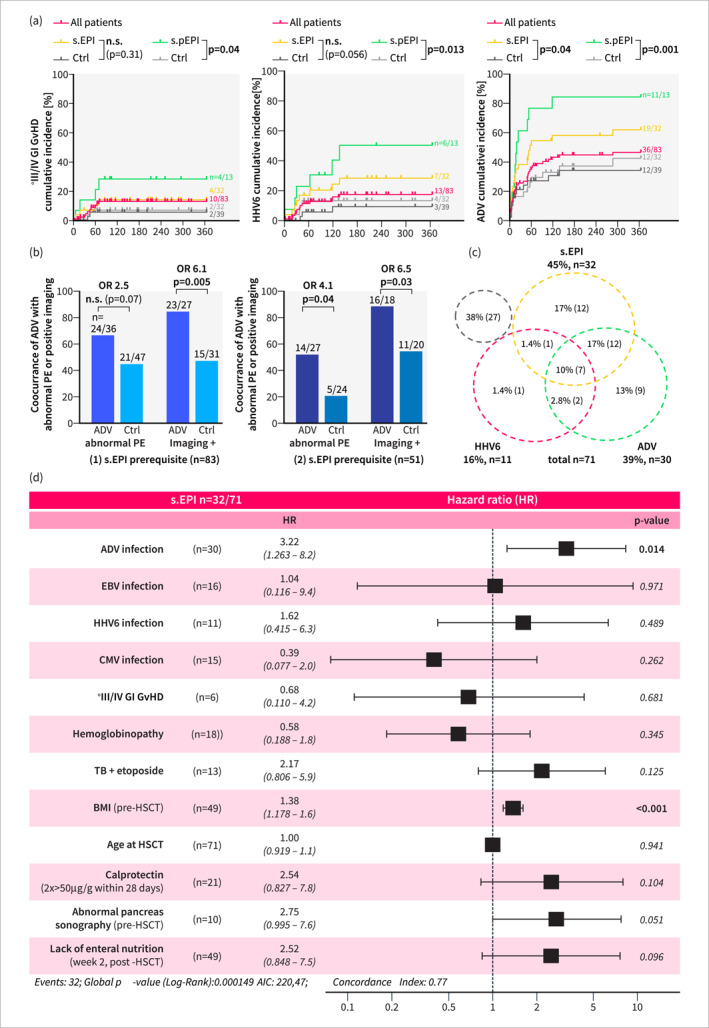
Adenovirus infection is an important risk factor for developing s.(p)EPI post‐HSCT. (a) Cumulative incidence of grade III/IV gut GvHD, human herpes virus 6 (HHV6) and adenovirus (ADV) infection in all patients, s.(p)EPI‐ and control groups. (b) Comparison of occurrence rates of abnormal PE levels or positive pancreatic imaging findings between patients with ADV infection and control patients (patients without ADV infection). Analyses were conducted using the two general cohorts from secondary endpoint analysis (1) and (2) (Figure 1): All patients with sufficient PE measurements for s.EPI group allocation ((1) s.EPI prerequisite, *n* = 83) or patients with sufficient PE measurements for s.pEPI group allocation ((2) s.pEPI prerequisite, *n* = 51). (c) Overlap in the cumulative incidence of s.(p)EPI with HHV6 and/or ADV infection post‐HSCT of all patients with sufficient PE measurements and pancreas imaging for s.EPI group allocation (*n* = 71). (d) Time‐dependent Cox regression analysis output depicting hazard ratios and *p* values of different possible s.EPI risk factors. Only patients with sufficient PE measurements and pancreas imaging for s.EPI group allocation (*n* = 71/83) were included. ADV: adenovirus, s.(p)EPI: suspected (prolonged) exocrine pancreas insufficiency, Ctrl: control group, d/w: days/weeks after HSCT, GvHD: graft‐versus‐host‐disease, HHV6: human herpes virus 6, HSCT: hematopoietic stem cell transplantation, PE: pancreas elastase, n: sample size. Statistical analysis: (a): Kaplan‐Meyer statistics and log‐rank test. (b): Fisher’s exact test. (d): Multivariable regression analysis. n.s.: not significant.

We observed a median onset of EPI within the first week (range: −2–6) for all s.EPI patients, with a median duration of 5 weeks (range: 2–47). Patients with s.pEPI exhibited a median onset in the third week (range: 1–15), lasting a median of 15 weeks (range: 8–47) (Figure 3a, Table S2 and S3). Regarding early immune reconstitution and survival outcomes, no significant differences were observed between s.(p)EPI and control patients during the trial period (Figure S3 and S5A, Table S2 and S3). However, to address immortal time bias and the short median observation period in the s.pEPI group (227 vs. 319 days), we extended follow‐up for up to 3 years for survival analysis of s.pEPI‐ and respective control patients. Here, we observed an increased non‐relapse mortality in the s.pEPI group (Hazard ratio (HR) 4.9, 95%CI [0.9, 28.2]) (Figure S3C). Additionally, while s.pEPI resolved in 5 patients within the first year post‐HSCT, 4 patients (31%) died before s.pEPI resolved. One patient (9%) exhibited pathological PE levels for more than 2 years post‐HSCT and showed ultrasound signs of pancreatic atrophy. In total, 3 s.pEPI cases showed ultrasound signs of pancreatic atrophy (i.e., severely reduced size). Examining other EPI‐associated complications s.(p)EPI patients experienced prolonged parenteral nutrition duration (s.EPI: median 92 vs. 45 days, s.pEPI: 101 vs. 68 days), extended initial HSCT hospitalization (s.EPI: 73 vs. 43 days, s.pEPI: 117 vs. 50 days), and substantial weight loss (Figure 3c–e). The s.pEPI subgroup experienced ongoing weight loss until d365, with a median loss of 20% body weight, compared to 2% gain in controls (Figure 3e, Figure S4G, Table S2 and S3). Moreover, appetite loss and delayed transition to a normal diet were more prevalent in the s.(p)EPI groups (Figure S4). Despite non‐significant differences in insulin or C‐peptide levels, s.(p)EPI patients exhibited higher glucose levels, a greater proportion with glucose more than 150 mg/dL, and a correlation between low PE levels and increased serum glucose (Figure S5B–F). Examining lipase profiles and the prevalence of elevated lipase levels (> 60 U/l) at different time points, we found no statistically significant differences between the s.(p)EPI and control groups. However, it is noteworthy that all four patients diagnosed with pancreatitis post‐HSCT were allocated to the s.EPI group (Figure S5G and H). Besides, s.(p)EPI group allocation was associated with a significantly higher incidence of secondary complications: S.(p)EPI‐patients experienced more severe adverse events (s.EPI: 24% (8/32), s.pEPI: 40% (5/13) versus. controls: 8%–9%), and needed more red blood cell (s.EPI: median 17 vs. 9, s.pEPI: median 30 vs. 12) and platelet transfusions (s.EPI: 28 vs. 11, s.pEPI: 73 vs. 12) (Figure 4a, Table S2 and S3).

### 4.3 Adenovirus Infection is a Strong Risk Factor and Possible Cause for EPI

During the trial period, none of the patients exhibited typical causes or risk factors for EPI as defined in the exclusion criteria (Figure 1) [4, 25]. Looking for other potential EPI risk factors and correlations, we found an increased occurrence of moderate/severe gastrointestinal (GI) GvHD in the s.pEPI group (31% (4/13) versus 6% (2/32)), as well as increased viral infection rates, notably systemic and intestinal ADV (s.EPI: 59% (19/32) versus 31% (12/39), s.pEPI: 85% (11/13) versus 38% (12/32)) and human herpesvirus 6 (HHV6) (s.EPI: 25% (8/32) % versus 8% (3/39), s.pEPI: 46% (6/13) versus 12.5% (4/32)) (Figure 4a, Table S2 and S3). In line with this, we observed higher stool calprotectin levels and a correlation between low PE values and high calprotectin levels (Figure S6). During GI GvHD, HHV6 and ADV sub‐group analysis, we observed a positive odds ratio (OR) for the occurrence of pathological PE values. By abdominal ultrasound, however, we found a significantly increased rate of abnormal pancreas sonography only in ADV patients (OR > 6), none in GI GvHD patients (OR 0.6–1) and only a weak correlation in HHV6 patients (OR 2.3–3.9) (Figure 4b, Figure S7). Supporting the hypothesis that EPI is especially associated with ADV, we found that all GI GvHD patients and 88% (7/8) of HHV6 patients who developed EPI had a history of ADV infection (Figure 4c). To validate these results, we conducted a time‐dependent multivariable risk factor analysis. Although not reaching statistical significance, positive hazard ratios (HR 1.5–3) were observed for HHV6 infection, total body irradiation as part of the conditioning regimen, abnormal pancreas imaging pre‐HSCT, abnormal calprotectin levels and full parenteral nutrition in week 2 post‐HSCT. Abnormal pancreas imaging, present in 13% (10/79) of patients across various malign and benign diseases, was considered a marker of potential pre‐existing pancreatic damage. In contrast, two factors emerged as significant independent risk factors for s.EPI group allocation: higher pre‐transplant BMI (HR 1.38) and ADV infection (HR 3.5). Notably, 75% (6/8) of s.pEPI patients with prolonged EPI despite enteral nutrition had a history of ADV preceding EPI onset (Figure 4d). To confirm the independence of ADV as a risk factor, we examined its co‐occurrence with other viral infections. While s.EPI patients demonstrated a higher cumulative incidence of viral infections, only a non‐significant proportion (3/12) of those who showed abnormal PE following ADV infection had concurrent infections with EBV, CMV, or HHV6 (Figure S8).

## 5 Discussion

This study reveals a significant incidence of exocrine pancreatic insufficiency post‐HSCT and demonstrates the diagnostic value of PE screening and pancreatic ultrasound. Fecal PE measurement shows high sensitivity and specificity in high‐risk cohorts, though borderline PE levels (150–250 μg/g), full parenteral nutrition and diarrhea may lead to false positives [4, 14, 17, 23]. Current guidelines recommend PE re‐testing and clinical symptoms to confirm EPI and complementary cross‐sectional imaging (computer tomography, magnetic resonance imaging or endoscopic ultrasound) to investigate potential causes [4, 5, 18]. Additionally, while cross‐sectional imaging is not recommended for direct EPI diagnosis, several studies have demonstrated correlations between specific pancreatic abnormalities, such as atrophy, and EPI [4, 5, 26, 27]. To reduce risks in our immunosuppressed pediatric population, we utilized non‐invasive transabdominal ultrasound, which has high diagnostic accuracy for pancreatic lesions [21]. As EPI‐associated clinical symptoms overlapped significantly with common post‐HSCT complications, they could not be reliably used to identify EPI in our cohort. Instead, we based the identification of patients with suspected EPI on PE levels and imaging findings. While the possibility of false positive misclassification cannot be completely ruled out, multiple indicators strongly support the presence of clinically relevant prolonged EPI in a substantial subset of patients identified through this approach: (1) Multiple pathological PE measurements and a significant proportion of PE levels < 100 µg/g were found in s.(p)EPI patients. (2) Similar proportional distribution of liquid samples was observed in all patients, regardless of group allocation, suggesting that diarrhea alone cannot explain the differences in PE levels. (3) Abnormal pancreatic imaging correlated highly with abnormal PE levels and showed pathological changes in the pancreas in all s.(p)EPI patients which cannot be explained by diarrhea or lack of enteral nutrition (Figure 2f). (4) s.pEPI patients showed severe weight‐loss during the first year after HSCT. (5) Individual s.pEPI patient analysis could exclude lack of enteral nutrition and diarrhea as confounders for abnormal PE values over periods longer than 4 weeks in 80% (8/10) of these patients. The study, however, recognizes the uncertainty regarding EPI’s contribution to secondary complications post‐HSCT, with both reverse causative and co‐occurrence scenarios plausible. While it is highly unlikely, that EPI is responsible for the increased 3‐year mortality observed in s.pEPI patients, it may contribute to multimorbidity. Besides, this is the first time a potential common cause has been identified, that could explain previously unclear cases of weight loss and growth retardation post‐HSCT [2]. Further research, ideally through randomized controlled trials applying PERT, is needed to determine and mitigate EPI’s exact effects. Additionally, the findings of severe pancreatic atrophy and ongoing EPI beyond two years post‐HSCT suggest a risk of permanent pancreatic damage.

Mechanistically, EPI post‐HSCT most likely results from the accumulation of multiple risk factors. Organ and mucosal damage resulting from chemotherapy and radiation therapy during conditioning, in combination with fasting, may contribute to early drops in PE levels, especially in patients with pre‐existing organ damage. Additionally, persistent inflammatory damage from mucositis, viral infections like ADV, or GI GvHD could increase the risk for prolonged damage further, consistent with evidence linking inflammatory bowel diseases and celiac disease to EPI [28, 29]. The strong contribution of ADV infection to EPI in our cohort aligns with studies associating ADV with pancreatic damage, which is more severe and persistent in immunosuppressed patients [8, 9, 10, 30]. Finally, these findings have significant implications beyond pediatric populations and HSCT, extending to other immunosuppressed and adult populations, including individuals with HIV, primary immunodeficiencies, or those undergoing systemic immunosuppressive therapy for autoimmune diseases. Such populations are highly susceptible to ADV and may experience underdiagnosed EPI [29, 31, 32]. Similar mechanisms likely contribute to EPI development in these diverse groups, emphasizing the need for vigilance and proactive management.

To improve outcomes and mitigate EPI post‐HSCT, we propose: (1) pre‐screening of patients before HSCT for pre‐existing pancreas damage using ultrasound and PE level testing to identify high‐risk patients and (2) implementation of routine fecal PE monitoring, with prompt initiation of PERT when clinically relevant EPI is suspected. While additional diagnostic tests could confirm diagnosis, they are not universally available and have limitations [4, 5, 17]. In contrast, PERT is well‐tolerated, readily accessible, and backed by strong evidence for its efficacy [33, 34, 35]. Early intervention with PERT is critical as EPI‐associated complications—such as weight loss, and increased hospitalization duration—pose serious risks, especially in young children, and contribute to increased healthcare costs and reduced quality of life. Parenteral nutrition alone is insufficient when underlying EPI is present. To that end, our study contributes to ongoing research on the benefits of earlier enteral nutrition post‐HSCT [36, 37]. As fat‐soluble vitamin uptake improves only partially upon PERT initiation, regular controls and parenteral substitution are recommended along with concomitant proton pump inhibitor therapy in cases of persistent malabsorption despite high dose PERT [3, 5, 33]. (3) Transabdominal ultrasound can help evaluate pancreatic damage and its complementary diagnostic role in immunocompromised patients warrants further investigation. (4) Other causes and risk factors for EPI, such as diabetes, inflammatory bowel diseases, or subclinical genetic predispositions like mild cystic fibrosis, should be investigated [4, 25]. (5) Given the strong association between ADV infection and EPI, early and aggressive treatment of ADV is warranted. This includes initiating antiviral therapy (e.g. using cidofovir or brincidofovir) even in cases where ADV is detected solely in feces. Consideration should also be given to the early use of allogeneic ADV‐specific T cells [38].

In conclusion, this study demonstrates the diagnostic and prognostic value of fecal PE measurements and pancreatic imaging, suggesting a high incidence of EPI following HSCT. ADV infection is identified as an important risk factor. This study emphasizes the need for increased awareness of the underdiagnosed complication EPI and advocates for regular monitoring of exocrine pancreas function. Early intervention strategies and aggressive management of ADV infections may improve patient outcomes post‐HSCT and reduce the health economic burden associated with EPI. Further research is required to refine these recommendations and explore their applicability in other immunosuppressed populations.

## Author Contributions

M.L. conceptualized the study, collected, curated, and visualized the data, conducted the formal analysis, and prepared the manuscript. A.M. performed the literature review, collected and analyzed data, and prepared the manuscript. J.S. performed ultrasound imaging, contributed representative images, and reviewed the manuscript. L.A. verified data, performed multivariable risk factor analysis, and reviewed the manuscript. S.C., F.Z., L.O., and A.K. conducted patient care and enrollment, participated in data discussions, and reviewed the manuscript. H.E.D., H.v.B., A.E., P.L., P.B., A.E., and A.v.S. participated in designing the study, discussing the data, validating findings, and reviewing the manuscript. A.P, contributed data and reviewed the manuscript. J.H.S. supervised the study, reviewed the data, results, and manuscript. Data were accessed and verified by M.L., A.M., L.A., and J.H.S. All authors had full access to all the data in the study, reviewed the manuscript, and had final responsibility for the decision to submit for publication.

## Conflicts of Interest

The authors declare no conflicts of interest.

## Declaration of Generative AI and AI‐Assisted Technologies in the Writing Process

During the preparation of this work, the authors used ChatGPT v. 4.0 (OpenAI) for proofreading of the manuscript. After using this tool, the authors reviewed and edited the content as needed and take full responsibility for the content of the publication.

## Supporting information

Supporting Information S1

## Data Availability

All aggregated data used for analysis of the study cohort are available in the manuscript, tables, figures, or supplemental data collection. Raw data collected for this study and detailed test statistics will be made available after formal request and approval by the corresponding author and upon approval of a formal data use agreement. All de‐identified data can be provided. The study protocol, statistical analysis plan, and informed consent form can also be provided with restrictions to academic use only. Raw data and additional documents will be available upon publication for up to 24 months. Commercialization of these data, if provided, is prohibited.
