# Supplementary material for: High Incidence and Impact of Suspected Exocrine Pancreatic Insufficiency in Patients Post‐Hematopoietic Stem Cell Transplantation: A Single‐Center Prospective Observational Study
Source: United European Gastroenterol J. 2025 Feb 15;13(2):257–67. doi: 10.1002/ueg2.12769 (PMC11975613; doi:10.1002/ueg2.12769)
Supplement: Supplementary file 1 — Supporting Information S1 [file UEG2-13-257-s001.docx]

High Incidence and Impact of Suspected Exocrine Pancreatic Insufficiency in Patients Post-Hematopoietic Stem Cell Transplantation: A Single-Center Prospective Observational Study

**- Supplementary Data -**

**Content**

[Detailed Statistical Methods: 2](#_Toc188896694)

[Summary of general patient characteristics 2](#_Toc188896695)

[Causes of death 3](#_Toc188896696)

[Tables 4](#_Toc188896697)

[Table 1 - General patient characteristics 4](#_Toc188896698)

[Table 2 - Comparison of Study Cohorts - s.EPI 6](#_Toc188896699)

[Table 3 - Comparison of Study Cohorts - s.pEPI 9](#_Toc188896700)

[Supplementary Figures 12](#_Toc188896701)

[Figure S1 12](#_Toc188896702)

[Figure S2 14](#_Toc188896703)

[Figure S3 16](#_Toc188896704)

[Figure S4 18](#_Toc188896705)

[Figure S5 20](#_Toc188896706)

[Figure S6 22](#_Toc188896707)

[Figure S7 24](#_Toc188896708)

[Figure S8 26](#_Toc188896709)

# Detailed Statistical Methods:

- **Comparison of Cohorts**: Mann-Whitney U test for continuous data and Fisher’s exact test for binary data.
- **Cumulative Incidence Analysis**: Kaplan-Meier statistics and the log-rank test.
- **Comparison of Three or More Groups**: Non-parametric Kruskal-Wallis test with Dunn’s multiple comparisons test.
- **Time-Point Analysis**: Mixed-model test combined with Sidak’s multiple comparisons post hoc test to account for missing data.
- **Secondary Endpoints**: Inverse Kaplan-Meier curves for endpoints (e.g., independence from parenteral nutrition), analyzed using the Gehan-Breslow-Wilcoxon test.
- **Correlation Analysis**: Spearman correlation.
- **Multivariable Risk Factor Analysis**: Cox regression model with time-dependent covariates using the survival and survminer packages in R.

# Summary of general patient characteristics

Between December 14, 2020, and April 31, 2023, 88 pediatric and young adult patients underwent allogeneic HSCT at our institution. Five were excluded: one with pre-diagnosed exocrine pancreatic insufficiency and four who declined participation. The study cohort of 83 patients included 37 females (44·6%) and 46 males (55·8%). Age groups were 7 infants (8·4%), 28 toddlers aged 1-5 years (33·7%), 30 children aged 6-11 years (36·1%), 12 adolescents aged 12-17 years (14·5%), and 6 young adults up to 22 years (7·2%). Median BMI at transplantation was 16·4 kg/m² (range: 12·7 to 31·9). 71 patients (86%) received their first HSCT. Transplantations were evenly distributed between matched (57%) and mismatched, as well as between BM or PBMC sources (55%). Underlying disease types were 46% malignant and 54% benign, with 21 different diseases treated. The most prevalent were acute B-cell lymphoblastic leukemia (22%), sickle cell disease (15%), and beta thalassemia major (11%). A T cell receptor alpha/beta T cell- and CD19 B cell-depleted transplant was administered to 34 patients (42%). 67 patients (83%) received an immunosuppressive regimen with serotherapy during conditioning (anti-thymocyte globulin or alemtuzumab) and ciclosporin A plus mycophenolate mofetil post-transplantation for at least 80-100 days based on individual indications and GvHD risk. We observed a relapse rate of 6%, treatment-related mortality of 9·6%, and graft rejection of 6%. Each patient had a median of 8 PE measurements (range: 2 to 45). Ultrasound imaging after HSCT was performed on 57 patients (69%), with 40 (58%) showing at least one pathological finding in the pancreas **(Fig. S1, Table S1).**

# Causes of death

s.pEPI:

Patient #66 - d63: Gastrointestinal bleeding during episode of severe GI-GvHD

Patient #67 - d527: Circulatory/multi organ failure due to multi-organ invasive mycosis

Patient #77 - d142: Pulmonary hemorrhage caused by invasive lung mycosis

Patient #80 - d93: Multi-organ failure on basis of hepatitis E infection

s.pEPI control group:

Patient #15 - d202: Multi-organ failure due to liver VOD

Patient #56 - d65: Right ventricular failure due to pulmonal veno-occlusive disease (VOD)

others (missing data for group allocation):

Patient #12 - d67: Multi-organ failure due to cytokine release syndrome

Paitent #62 - d29: Multi-organ failure due to engraftment syndrome and potential sepsis

Patient #65 - d54: Multi-organ failure due to ADV infection

Patient #68 - d2: Multi-organ failure due to conditioning-related toxicity

Patient #69 - d29: Multi-organ failure due to liver VOD

# Tables

Data were collected from digital patient record managers SAP (SAP SE, Walldorf, Germany) and COPRA (COPRA System GmbH, Berlin, Germany).

Laboratory values with age-dependent normal ranges were provided by Labor Berlin (Labor Berlin - Charité Vivantes GmbH, Berlin, Germany).

| Table 1 - General patient characteristics | |
| --- | --- |
| **Characteristics** | **median (range) / n (%)** |
| Observation period [days] ^a^ | 365 (1 - 365) |
| Cumulative days observed [days] | 21157 |
| Number of transplantations Dec. 2020 - Jun. 2023  Number of excluded patients, n (%) | 88  5(5.7) |
| Sex, n (%) |  |
| Male | 46 (55.8) |
| Female | 37 (44.6) |
| Age at transplantation [years] ^a^ | 7 (0-22) |
| Patients per age group, n (%)  <1  1-5  6-11  12-17  18+ | 7 (8.4)  28 (33.7)  30 (36.1)  12 (14.5)  6 (7.2) |
| Bodyweight at transplantation [kg] ^a^ | 24.9 (4.8-129) |
| HSCT count, n (%) |  |
| 1 | 71(85.5) |
| 2 | 8 (9.6) |
| 3 | 4 (4.8) |
| Type of HSCT, n (%) |  |
| MUD | 29 (34.9) |
| MMUD | 14 (16.9) |
| MSD | 18 (21.7) |
| MMRD/haploidentical | 22 (26.5) |
| Source of HSC, n (%) |  |
| BM | 45 (54.9) |
| PBSC | 35 (42.7) |
| Cord blood | 1 (1.2) |
| Cord blood and BM | 1 (1.2) |
| Underlying disease type, n (%) |  |
| Benign | 45 (54.2) |
| Malignant | 38 (45.8) |
| HSCT indication, n (%) |  |
| Acute myeloid leukemia  Beta thalassemia major | 7 (8.4)  9 (10.8) |
| Chronic granulomatous disease (CGD) | 4 (4.8) |
| B-cell acute lymphoblastic leukemia | 18 (21.7) |
| Diamond-Blackfan anemia | 2 (2.4) |
| Fanconi anemia | 2 (2.4) |
| Galactosialidosis | 1 (1.2) |
| Granuloma | 1 (1.2) |
| Hemophagocytic lymphohistiocytosis (HLH) | 1 (1.2) |
| Juvenile myelomonocytic leukemia (JMML) | 2 (2.4) |
| Myelodysplastic syndrome (MDS) | 5 (6.0) |
| Myelosarkoma | 1 (1.2) |
| Nephroblastoma | 1 (1.2) |
| Neuroblastoma | 3 (3.6) |
| Osteopetrosis | 1 (1.2) |
| Severe aplastic anemia | 1 (1.2) |
| Severe congenital neutropenia (Kostmann syndrome) | 2 (2.4) |
| Sickle cell disease (SCD) | 12 (14.5) |
| Severe combined immunodeficiency (SCID) | 3 (3.6) |
| T-cell acute lymphoblastic leukemia | 6 (7.2) |
| Wiskott-Aldrich-syndrome (WAS) | 1 (1.2) |
| Graft manipulation, n (%) |  |
| None | 44 (54.3) |
| CD3/CD19 depletion + CD3 addback | 1 (1.2) |
| TCRab/CD19 depletion +/- CD3 addback | 34 (41.9) |
| CD34 selection + TCRab/CD19 depletion | 2 (2.5) |
| Blood group matching, n (%) |  |
| Match | 38 (49.4) |
| Mismatch (AB0 or Rh) | 39 (50.6) |
| HLA matching, n (%) |  |
| 5/10 | 22 (26.5) |
| 9/10 | 15 (18.1) |
| 10/10 | 33 (40.0) |
| 12/12 | 13 (15.7) |
| Most common conditioning therapy, n (%) |  |
| Fludarabine, treosulfan, thiotepa, | 22 (26.5) |
| Total body irradiation, etoposide | 15 (18.1) |
| Conditioning regimen with serotherapy (i.e. anti-thymocyte globulin or alemtuzumab) + Ciclosporin A, Mycophenolate Mofetil, n (%) | 67 (82.7) |
| Transplanted CD34+ cells [x10^6^/kg BW] | 6.6 (1.4 – 79.9) |
| Transplanted CD3+ cells [x10^6^/kg BW] | 39.3 (1.4 – 232.9) |
| Drop out events: |  |
| Relapses, n (%) | 5 (6.0) |
| Non-relapse related deaths, n (%) | 8 (9.6) |
| Non-relapse graft rejections, n (%) | 5 (6.0) |
| Lost to follow-up, n (%) | 1 (1.2) |
| End of study period, n (%) | 21 (25.3) |
| Cause of death, n (%) |  |
| Multi organ failure | 6 (75.0) |
| Gastrointestinal bleeding | 1 (12.5) |
| Pulmonal veno-occlusive disease (VOD) | 1 (12.5) |
| **B - EPI related data** | **median (range) / n (%)** |
| PE measurements per patients ^a^ | 8 (2-45) |
| At least 2 PE measurements in a period of 7 to 28 days with at least 7 days between 2 measurements, n (%) | 83 (100) |
| At least 4 PE measurements in a period of at least 8 weeks with min. 7 days to max. 8 weeks between two measurements, n (%) | 51 (61.4) |
| Patients with pathological pancreas ultrasound imaging before conditioning | 10/79 (12.7) |
| Patients with pancreas ultrasound imaging post-HSCT, n (%)  0  1  2  ≥3 | 35(42.2)  22 (26.5)  17 (20.5)  7 (8.4) |
| Patients with pathological pancreas imaging findings post-HSCT, n (%) | 36 (75) |
|  |  |

^a^ Median (minimum - maximum)

*EPI: Exocrine pancreatic insufficiency, HSCT: Hematopoietic stem cell transplantation, MUD: Matched unrelated donor, MSD: Matched sibling donor, MMRD: Mismatch related donor, MRD: Matched related donor, HSC:* Hematopoietic stem cell, *BM: Bone marrow, PBSC: Peripheral blood stem cells, BW: body weight., PE: Pancreas elastase.*

| Table 2 - Comparison of Study Cohorts - s.EPI | |  | |  | |  | |
| --- | --- | --- | --- | --- | --- | --- | --- |
| **A - General Characteristics** | **s.EPI**  **n=32** | **Ctrl**  **n=39** | **p-value ^a^** | | **Effect size ^b^** | |  |
| Observation period [days] ^c^ | 279 (29-365) | 365(2-365) | 0.81 | | -86 | |  |
| Cumulative days observed [days] | 7879 | 9701 |  | |  | |  |
| Sex, n (%) |  |  |  | |  | |  |
| Male | 18 (56.3) | 22 (56.4) | >0.99 | | 1.0 | |  |
| Female | 14 (43.8) | 17 (43.6) |  | |  | |  |
| Age at transplantation [years] ^c^ | 9 (0 - 22) | 6 (0 - 21) | 0.08 | | 3 | |  |
| Patients per age group, n (%)  <1  1-5  6-11  12-17  18+ | 3 (9.4)  9 (28.1)  11 (34.4)  7 (21.9)  2 (6.3) | 3 (7.7)  14 (35.9)  16 (41.0)  4 (10.3)  2 (5.1) | >0.99  0.61  0.63  0.20  >0.99 | | 1.1  0.8  0.9  1.5  1.1 | |  |
| Bodyweight at transplantation [kg] ^c^ | 30.7 (4.9-129) | 19.5 (4.8-55) | **0.02** | | **+11** | |  |
| HSCT count, n (%) |  |  |  | |  | |  |
| 1 | 28 (87.5) | 32 (82.0) | 0.74 | | 1.3 | |  |
| 2 | 1 (3.1) | 6 (15.4) | 0.12 | | 0.3 | |  |
| 3 | 3 (9.4) | 1 (2.6) | 0.32 | | 1.7 | |  |
| Type of HSCT, n (%) |  |  |  | |  | |  |
| MUD  MMUD | 13 (40.6)  5 (15.6) | 9 (23.1)  6 (15.4) | 0.13  >0.99 | | 1.5  1.0 | |  |
| MSD | 5 (15.6) | 13 (33.3) | 0.11 | | 0.5 | |  |
| MMRD/haploidentical | 9 (28.1) | 11 (28.2) | >0.99 | | 1.0 | |  |
| Source of HSC, n (%) |  |  |  | |  | |  |
| BM | 18 (56.3) | 21 (55.3) | >0.99 | | 1.0 | |  |
| PBSC  Cord blood  Cord blood and BM | 14 (43.8)  0 (0)  0 (0) | 15 (39.5)  1 (2.6)  1 (2.6) | 0.81  >0.99  >0.99 | | 1.1  -  - | |  |
| Underlying disease type, n (%) |  |  |  | |  | |  |
| Benign | 17 (53.1) | 22 (56.4) | 0.81 | | 0.9 | |  |
| Malign | 15 (46.9) | 17 (43.6) |  |  |  | |  |
| HSCT indication, n (%) |  |  |  | |  | |  |
| Acute myeloid leukemia | 2 (6.3) | 5 (12.8) | 0.45 | | 0.6 | |  |
| Beta thalassemia major | 2 (6.3) | 6 (15.4) | 0.28 | | 0.5 | |  |
| Chronic granulomatous disease (CGD) | 2 (6.3) | 2 (5.1) | >0.99 | | 1.1 | |  |
| B-cell acute lymphoblastic leukemia | 9 (28.1) | 6 (15.4) | 0.25 | | 1.5 | |  |
| Diamond-Blackfan anemia | 2 (6.3) | 0 (0) | 0.20 | | 2.3 | |  |
| Fanconi anemia  Granuloma | 1 (3.1)  0 (0) | 1 (2.6)  1 (2.6) | >0.99  >0.99 | | 1.1  - | |  |
| Hemophagocytic lymphohistiocytosis (HLH) | 1 (3.1) | 0 (0) | 0.45 | | 2.26 | |  |
| Juvenile myelomonocytic leukemia (JMML) | 0 (0) | 2 (5.1) | 0.50 | | - | |  |
| Myelodysplastic syndrome (MDS) | 2 (6.3) | 2 (5.1) | >0.99 | | 1.1 | |  |
| Myelosarkoma | 1 (3.1) | 0 (2.6) | 0.45 | | 2.3 | |  |
| Nephroblastoma | 1 (3.1) | 1 (2.2) | >0.99 | | 1.1 | |  |
| Neuroblastoma | 1 (3.1) | 0 (0) | 0.45 | | 2.3 | |  |
| Osteopetrosis | 1 (3.1) | 0 (0) | 0.45 | | 2.3 | |  |
| Severe congenital neutropenia | 0 (0) | 2 (5.1) | 0.50 | | - | |  |
| Sickle cell disease (SCD) | 4 (12.5) | 6 (15.4) | >0.99 | | 0.9 | |  |
| Severe combined immunodeficiency (SCID) | 1 (3.1) | 2 (5.1) | >0.99 | | 0.7 | |  |
| T-cell acute lymphoblastic leukemia | 2 (6.3) | 2 (5.1) | >0.99 | | 1.1 | |  |
| Wiskott-Aldrich-syndrome (WAS) | 0 (0) | 1 (2.2) | >0.99 | | - | |  |
| Graft manipulation, n (%) |  |  |  | |  | |  |
| None | 16 (51.6) | 22 (56.4) | 0.81 | | 0.9 | |  |
| CD3/CD19 depletion + CD3 addback | 0 (0) | 1 (2.6) | >0.99 | | - | |  |
| TCRab/CD19 depletion +/- CD3 addback | 13 (42) | 16 (61.8) | >0.99 | | 1.0 | |  |
| CD34 selection + TCRab/CD19 depletion | 2 (6.5) | 0 (0) | 0.20 | | 2.3 | |  |
| Blood group (recipient/donor), n (%) |  |  |  | |  | |  |
| Match | 14 (46.7) | 20 (57.1) | 0.46 | | 0.8 | |  |
| Missmatch (AB0 or Rh) | 16 (53.3) | 15 (42.9) |  | |  | |  |
| HLA matching, n (%) |  |  |  | |  | |  |
| 5/10 | 8 (25.8) | 11 (28.2) | >0.99 | | 0.9 | |  |
| 9/10 | 4 (12.9) | 5 (12.8) | >0.99 | | 1.0 | |  |
| 10/10 | 15 (48.4) | 12 (30.8) | 0.15 | | 1.5 | |  |
| 12/12  9-10/10 | 3 (9.7)  1 (3.2) | 9 (23.1)  2 (5.1) | 0.20  >0.99 | | 0.5  0.8 | |  |
| Most common conditioning regimen, n (%) |  |  |  | |  | |  |
| Fludarabine, treosulfan, thiotepa, | 8 (25.0) | 11 (28.2) | 0.80 | | 0.9 | |  |
| total body irradiation, etoposide | 10 (31.3) | 3 (7.7) | **0.01** | | **2.0** | |  |
| Conditioning regimen containing serotherapy (i.e. anti-thymocyte globulin or alemtuzumab), n (%) | 26 (86.7) | 31 (79.5) | 0.53 | | 1.4 | |  |
| Transplanted CD34+ cells [x10^6^/kg BW] ^c^ | 7.4 (1.4-79.9) | 7.4 (1.7-38.8) | 0.69 | | 0 | |  |
| Transplanted CD3+ cells [x10^6^/kg BW] ^c^ | 24.9 (1.4 -134.6) | 46.8 (7.4-201.1) | 0.14 | | -22 | |  |
| Drop out events: |  |  |  | |  | |  |
| Relapses, n (%) | 3 (9.4) | 1 (2.6) | 0.32 | | 1.7 | |  |
| Non-relapse related deaths, n (%) | 4 (12.5) | 4 (10.3) | >0.99 | | 1.1 | |  |
| Non-relapse graft rejections, n (%) | 0 (0) | 5 (12.8) | 0.07 | | - | |  |
| Cause of death, n (%) |  |  | 0.13 | |  | |  |
| Multi organ failure, n (%) | 2 (50.0) | 4 (100) | 0.43 | | 0.3 | |  |
| Gastrointestinal bleeding, n (%) | 1 (25.0) | 0 (0) | >0.99 | | 2.3 | |  |
| Pulmonal veno-occlusive disease, n (%) | 1 (25.0) | 0 (0) | >0.99 | | 2.3 | |  |
| **B – s.EPI related data** | **s.EPI**  **n=32** | **Ctrl**  **n=39** | **p-value ^a^** | | **Effect size ^b^** | |  |
| PE-measurements per patients ^c^ | 11 (4-36) | 7 (2-26) | **0.03** | | **+4** | |  |
| Pancreas ultrasound imaging from w-1 onward, n (%)  1  2  3  4  ≥5 | 13 (46.4)  10 (35.7)  1 (3.6)  3 (10.7)  1 (3.6) | 15 (53.6)  10 (35.7)  2 (7.1)  1 (3.6)  0 (0) | 0.79  >0.99  >0.99  0.61  >0.99 | | 0.9  1.0  0.7  1.6  2.0 | |  |
| Patients with pathological pancreas ultrasound imaging, n (%) | 28 (100) | 10 (38.5) | **<0.0001** | |  | |  |
| Patients with PE<200 µg/g in solid stool samples, n (%) | 9 (45.0) | 2 (9.5) | **0.01** | | **2.2** | |  |
| s.EPI onset [week after HSCT] ^c, d^ | 1 (-2 - 6) |  |  | |  | |  |
| s.EPI duration [weeks] ^c, e^ | 5 (2-47) |  |  | |  | |  |
| Pancreas enzyme substitution therapy, n (%)^f^ | 6 (18.8) | 0 (0) | **0.006** | | **2.5** | |  |
| Insulin substitution, n (%) | 6 (18.8) | 0 (0) | **0.01** | | **2.5** | |  |
| Body weight d100 vs before HSCT [%] ^c^ | -6.6 (-24 - 87.1) | 0 (-16.7 - 24) | **0.006** | | **-6.6** | |  |
| Body weight d365 vs before HSCT [%] ^c^ | - 2.1 (-20.8 – 58.3) | 11.8 (-8.9 - 72.8) | **0.026** | | **-14** | |  |
| Z-Score d100 vs. before HSCT ^c^ | -0.9 (-1.8 - 1.6) | -0.3 (-1.5 - 1.3) | **0.02** | | **-0.6** | |  |
| Discolored or green stool, n (%) | 25 (80.7) | 31 (79.5) | >0.99 | | 1.0 | |  |
| Diarrhea during HSCT-hospitalization, n (%) | 30 (96.8) | 37 (94.9) | >0.99 | | 1.3 | |  |
| Diarrhea episodes, n ^c^ | 5 (0-13) | 3 (0-23) | **0.01** | | **+5** | |  |
| Parenteral nutrition duration [days] ^c^ | 60 (13-365) | 37 (1-365) | 0.15 | | +37 | |  |
| No or only little liquid food intake in week 2 after HSCT | 27 (84.4) | 22 (56.4) | **0.02** | | **1.5** | |  |
| **C - Immune reconstitution** | **s.EPI**  **n=32** | **Ctrl**  **n=39** | **p-value ^a^** | | **Effect size ^b^** | |  |
| B cell count at d365 [/nl] ^c^ | 0.6 (0-1.8) | 0.4 (0-1.3) | 0.64 | | +0.2 | |  |
| Neutrophil count >500/µl [days after HSCT] ^c^ | 17 (9-38) | 19 (0- 1) | 0.69 | | -2 | |  |
| Neutrophil count >1000/µl [d] ^c^ | 19 (9-60) | 20 (0-32) | 0.83 | | -1 | |  |
| Platelet count >50000/µl [days after HSCT] ^c^ | 17 (2-152) | 29 (8-259) | 0.09 | | -12 | |  |
| Lymphocyte count at d365 [/nl] ^c^  CD3+ count at d365 [/nl] ^c^  CD4+ count at d365 [/nl] ^c^  CD8+ count at d365 [/nl] ^c^ | 2.3 (0.3-6.2)  1.5 (0.1-3.1)  0.6 (0.1-3.1)  0.7 (0-3.2) | 2.0 (0.2-4.3)  1.3 (0.1-2.9)  0.5 (0.1-1.9)  0.5 (0.1-1.9) | 0.25  0.38  0.59  0.43 | | +0.3  +0.2  +0.1  +0.2 | |  |
| Total chimerism at d365 [%] ^c^  CD34 chimerism d365 [%] ^c^ | 100 (98-100)  100 (75-100) | 100 (58-100)  100 (64-100) | 0.74  0.55 | | 0  0 | |  |
| **D - Secondary complications** | **s.EPI**  **n=32** | **Ctrl**  **n=39** | **p-value ^a^** | | **Effect size ^b^** | |  |
| Duration of hospitalization for HSCT [days] ^c^ | 73 (33-225) | 43 (17-236) | **0.001** | | **+30** | |  |
| Cumulative re-hospitalization duration [days] ^c^ | 16 (0-83) | 10 (0-75) | 0.17 | | +6 | |  |
| Survived severe adverse events, n (%) ^g^ | 8 (23.5) | 3 (7.7) | 0.1 | | 1.7 | |  |
| Stem cell boost, n (%) | 3 (9.7) | 5 (12.8) | >0.99 | | 0.8 | |  |
| Donor lymphocyte infusions, n (%) | 2 (6.5) | 5 (12.8) | 0.45 | | 0.6 | |  |
| Viral infection (>2000 copies/ml in blood), n (%) | 28 (87.5) | 25 (64.1) | **0.03** | | **2.4** | |  |
| Number of viral infections with >2000 copies/ml blood per patient, n (%) |  |  |  | |  | |  |
| 1 | 6 (18.8) | 8 (20.5) | >0.99 | | 0.9 | |  |
| 2 | 5 (15.6) | 8 (20.5) | 0.76 | | 0.8 | |  |
| 3 | 7 (21.9) | 5 (12.8) | 0.35 | | 1.4 | |  |
| 4  5  ≥6 | 3 (9.4)  1 (3.1)  6 (18.8) | 2 (5.1)  1 (2.6)  1 (2.6) | 0.65  >0.99  **0.04** | | 1.4  1.1  **2.1** | |  |
| EBV infection (>2000 copies/ml blood), n (%) | 9 (28.1) | 7 (18.0) | 0.40 | | 1.3 | |  |
| ADV infection (>2000 copies/ml blood), n (%) | 14 (43.8) | 8 (20.5) | **0.04** | | **1.7** | |  |
| ADV finding in feces n (%) | 15 (46.9) | 12 (30.8) | 0.22 | | 1.4 | |  |
| CMV infection (>2000 copies/ml blood), n (%) | 8 (25.0) | 8 (20.5) | 0.77 | | 1.1 | |  |
| HHV6 infection (>2000 copies/ml blood), n (%) | 4 (12.5) | 0 (0) | **0.04** | | **-** | |  |
| HHV6 finding in feces, n (%) | 6 (37.5) | 3 (16.7) | 0.25 | | 1.7 | |  |
| Cumulative occurrence of HHV6, EBV and/or CMV | 21 (65.6) | 13 (33.3) | **0.009** | | **2** | |  |
| BKV infection (>2000 copies/ml blood), n (%) | 14 (66.7) | 7 (33.3) | 0.15 | | 1.5 | |  |
| BKV finding in urin, n (%) | 17 (56.7) | 9 (37.5) | 0.18 | | 1.4 | |  |
| Virus-specific T cells, n (%) | 8 (25.0) | 11 (28.2) | 0.79 | | 0.9 | |  |
| Neutropenia relapses <500/µl blood ^c, h^ | 0 (0 - 4) | 0 (0 - 4) | 0.99 | | 0 | |  |
| Intravenous antibiotic treatment initiaitions ^c^ | 2 (1-6) | 2 (1-5) | 0.10 | | 0 | |  |
| Positive blood cultures, n (%) | 14 (43.8) | 13 (33.3) | 0.46 | | 1.3 | |  |
| Systemic antimycotic treatment in therapeutic dose, n (%) | 30 (96.8) | 26 (76.5) | **0.03** | | **4.8** | |  |
| Relevant acute GvHD, n (%) ^i^ | 9 (34.6) | 5 (20.8) | 0.52 | | 1.3 | |  |
| Relevant acute GI GvHD, n (%) ^i^ | 4 (12.5) | 2 (5.1) | 0.35 | | 1.4 | |  |
| Moderate - severe chronic GI GvHD, n (%) | 3 (10.7) | 1 (4.0) | 0.6 | | 1.4 | |  |
| Cumulative systemic steroid treatments ^c^ | 1 (0-5) | 0 (0-3) | 0.09 | | +1 | |  |
| Extra-corporal photopheresis (ECP), n (%) | 3 (9.7) | 3 (7.7) | >0.99 | | 1.1 | |  |
| Vedolizumab, basiliximab and/or infliximab, n (%) | 13 (41.9) | 12 (30.8) | 0.45 | | 1.3 | |  |
| Pancreatitis diagnosis, n (%) | 4 (12.5) | 0 | **0.03** | | 2.4 | |  |
| Red blood cell concentrates per patient, n ^c^ | 17 (2-76) | 9 (1-64) | **0.004** | | **+8** | |  |
| Red blood cell concentrates transfused (total), n | 723 | 575 |  | |  | |  |
| Platelet concentrates per patient, n ^c^ | 28 (3-295) | 11 (1-110) | **0.0088** | | **+17** | |  |
| Platelet concentrates transfused (total), n | 1682 | 698 |  | |  | |  |
|  |  |  |  | |  | |  |

^a^ To compare cohorts the Mann-Whitney U rank test was used for continuous data and the Fishers exact test for binary data. GraphPad PRISM 8 & 9 (GraphPad Software, San Diego, USA).

^b^ For comparisons of continuous data the *difference of median* and for comparisons of binary data the *relative risk* are given as an indicator of the effect size.

^c^ Median (range).

^d^ s.EPI onset was defined as the week of the first PE level <200µg/g within the s.EPI defining time period

^e^ s.EPI end point was defined as last PE level <200µg/g of the defining time period with the following two consecutive measurements showing levels >200µg/g.

^f^ As PERT was not part of the study design important information for assessing its influence is missing. Symptoms directly before and after start were not documented in a systematic manner. Information on compliance and correct use is missing for most patients. Hence, it was not feasible to analyze the effect of PERT in our study further and its effect on the study cohort has to be interpreted with caution. Initiation happened at a mean of 128 days of s.(p)EPI group allocation PE level abnormality onset.

^g^ Oriented on common terminology criteria for adverse events (CTCAE) version 5.0 Grade 4: Complications that necessitated transfer to ICU, Catecholamines to prevent cardiac failure, dialysis or invasive ventilation.

^h^ New drop of neutrophil blood level below 1000/µl after previous regeneration of neutrophils (>1000/µl) for at least 7 days (independent of granulocyte colony stimulating factor treatment)

^i^ This includes every episode of acute GvHD for which patients received systemic treatment like steroids and/or extracorporeal photopheresis.

*s.EPI: suspected Exocrine pancreatic insufficiency, HSCT: Hematopoietic stem cell transplantation, MUD: Matched unrelated donor, MSD: Matched sibling donor, MMRD: Mismatch related donor, MRD: Matched related donor, HSC:* Hematopoietic stem cell, *BM: Bone marrow, PBSC: Peripheral blood stem cells, BW: body weight. EBV: Epstein-Barr virus, ADV: Adenovirus, CMV: Cytomegalovirus, HHV6: Human herpes virus 6, BKV: BK virus, PE: Pancreas elastase. GvHD: Graft versus host disease*

| Table 3 - Comparison of Study Cohorts - s.pEPI | |  |  |  |
| --- | --- | --- | --- | --- |
| **A - General Characteristics** | **s.pEPI**  **n=13** | **Ctrl**  **n=32** | **p-value ^a^** | **Effect size^b^** |
| Observation period [days] ^c^ | 227 (63-365) | 319 (49-365) | 0.55 | -92 |
| Sex, n (%) |  |  |  |  |
| Male | 8 (61.5) | 16 (50.0) | 0.53 | 1.4 |
| Female | 5 (38.5) | 16 (50.0) |  |  |
| Age at transplantation [years] ^c^ | 7 (0 - 16) | 7 (0 - 21) | 0.79 | 0 |
| Patients per age group, n (%)  <1  1-5  6-11  12-17  18+ | 1 (7.7)  4 (30.8)  6 (38.5)  3 (23.1)  0 (0) | 4 (12.5)  9 (28.1)  12 (37.5)  5 (15.6)  2 (6.3) | >0.99  >0.99  >0.99  0.67  >0.99 | 0.6  1.0  1.0  1.4  - |
| Bodyweight at transplantation [kg] ^c^ | 27.5 (7.9-129) | 25.3 (4.8-74) | 0.63 | +2.2 |
| HSCT count, n (%) |  |  |  |  |
| 1 | 11 (84.6) | 28 (87.5) | >0.99 | 0.8 |
| 2 | 1 (7.7) | 2 (6.3) | >0.99 | 1.2 |
| 3 | 1 (7.7) | 2 (6.3) | >0.99 | 1.2 |
| Type of HSCT, n (%) |  |  |  |  |
| MUD  MMUD | 4 (30.8)  3 (23.1) | 12 (37.5)  5 (15.6) | >0.99  0.67 | 0.8  1.4 |
| MSD | 4 (30.8) | 9 (29.0) | >0.99 | 1.1 |
| MMRD/haploidentical | 2 (15.4) | 6 (18.8) | >0.99 | 0.8 |
| Source of HSC, n (%) |  |  |  |  |
| BM | 9 (69.2) | 19 (61.3) | 0.74 | 1.3 |
| PBSC  Cord blood +/- BM | 4 (28.6)  0 (0) | 12 (38.7)  2 (5.2) | 0.74  >0.99 | 0.8  - |
| Malignancy, n (%) |  |  |  |  |
| Benign | 8 (61.5) | 16 (50.0) | 0.53 | 1.4 |
| Malign | 5 (38.5) | 16 (50.0) |  |  |
| HSCT indication, n (%) |  |  |  |  |
| Acute myeloid leukemia | 0 (0) | 5 (15.6) | 0.30 | - |
| Beta thalassemia major | 3 (23.1) | 3 (9.4) | 0.33 | 2 |
| Chronic granulomatous disease (CGD) | 1 (7.7) | 2 (6.3) | >0.99 | 1.2 |
| B-cell acute lymphoblastic leukemia | 3 (23.1) | 9 (28.1) | 0.73 | 0.8 |
| Diamond-Blackfan anemia | 2 (15.4) | 0 (0) | 0.08 | 3.9 |
| Louis-Bar syndrome with necrotizing  granuloma | 0 (0) | 1 (3.1) | >0.99 | - |
| Myelodysplastic syndrome (MDS) | 2 (15.4) | 2 (6.3) | 0.57 | 1.9 |
| Myelosarkoma | 0 (0) | 1 (3.1) | >0.99 | - |
| Nephroblastoma | 1 (7.7) | 1 (3.1) | 0.5 | 1.8 |
| Sickle cell disease (SCD) | 0 (0) | 4 (12.5) | 0.31 | - |
| Severe combined immunodeficiency (SCID) | 0 (0) | 3 (9.4) | 0.55 | - |
| T-cell acute lymphoblastic leukemia | 1 (7.7) | 0 (0) | >0.99 | - |
| Graft manipulation, n (%) |  |  |  |  |
| None | 7 (58.3) | 18 (50) | 0.74 | 1.3 |
| CD3/CD19 depletion + CD3 addback | 0 (0) | 1 (3.1) | >0.99 | - |
| TCRab/CD19 depletion +/- CD3 addback | 5 (38.5) | 10 (31.3) | >0.99 | 1.0 |
| CD34 selection + TCRab/CD19 depletion | 0 (0) | 1 (3.1) | >0.99 | - |
| Blood group (recipient/donor), n (%) |  |  |  |  |
| Match | 6 (50.0) | 13 (43.3) | 0.74 | 1.2 |
| Missmatch (AB0 or Rh) | 6 (50.0) | 17 (56.7) |  |  |
| HLA matching, n (%) |  |  |  |  |
| 5/10 | 2 (15.4) | 6 (18.8) | >0.99 | 0.8 |
| 9/10 | 3 (23.1) | 4 (12.5) | 0.39 | 1.6 |
| 10/10 | 6 (46.2) | 14 (43.8) | >0.99 | 1.1 |
| 12/12  9-10/10 | 2 (15.4)  0 (0) | 7 (21.9)  1 (3.1) | >0.99  >0.99 | 0.7  - |
| Most common conditioning therapy, n (%) |  |  |  |  |
| Fludarabine, treosulfan, thiotepa | 3 (23.1) | 9 (28.1) | 0.47 | 0.6 |
| total body irradiation, etoposide | 3 (23.1) | 10 (31.3) | 0.73 | 0.7 |
| Conditioning regimen containing serotherapy (i.e. anti-thymocyte globulin or alemtuzumab), n (%) | 12 (92.3) | 29 (90.6) | >0.99 | 1.2 |
| Transplanted CD34+ cells [x10^6^/kg BW] ^c^ | 5.5 (1.4-79.9) | 7.4 (1.4-42.3) | 0.61 | -1.9 |
| Transplanted CD3+ cells [x10^6^/kg BW] ^c^ | 22.6 (1.4-91.3) | 40.9 (2.7- 201) | 0.43 | -18.3 |
| Drop out events: |  |  |  |  |
| Relapses, n (%) | 0 (0) | 3 (9.4) | 0.55 | - |
| Non-relapse related deaths, n (%) | 1 (7.7) | 1 (3.1) | 0.5 | 1.8 |
| Cause of death, n (%) |  |  |  |  |
| Gastrointestinal bleeding, n (%) | 1 (100) | 0 (0) | 0.20 | - |
| Lung veno-occlusive disease (VOD), n (%) | 0 (0) | 1 (100) | >0.99 | - |
| **B – s.pEPI related data** | **s.pEPI**  **n=13** | **Ctrl**  **n=32** | **p-value** ^a^ | **Effect size^b^** |
| PE1-measurements per patients ^c^ | 14 (6-36) | 13 (6-26) | 0.65 | +1 |
| Pancreas ultrasound imaging from w-1 onward, n (%)  0  1  >1 | 0 (0)  3 (23)  10 (76.9) | 9 (28.1)  13 (40.6)  10 (31.3) | **0.04**  0.32  0.09 | -  0.5  2.4 |
| Patients with pathological pancreas ultrasound imaging per patient, n (%) | 13 (100) | 24 (72.7) | **0.01** | - |
| s.pEPI onset [weeks after HSCT] ^c, d^ | 3 (1 - 15) | - | **-** | - |
| s.pEPI duration [weeks] ^c, e^ | 15 (8-47) | - | - | - |
| Pancreas enzyme substitution therapy, n (%) ^f^ | 6 (46.2) | 1 (3.1) | **0.001** | **4.7** |
| Insulin substitution, n (%)^g^ | 3 (23.1) | 1 (3.1) | **0.07** | **3.1** |
| Body weight d100 vs. before HSCT [%] ^c^ | -11 (-24 - 25) | 1.6 (-15 - 24) | **0.02** | **-12.6** |
| Body weight d365 vs. before HSCT [%] ^c^ | -8.6 (-21 - 8.7) | 2.4 (-9.5 - 72.8) | **0.01** | **-11** |
| Z-Score d100 vs. before HSCT ^c^ | -1.0 (-1.3 - 1.3) | -0.3 (-1.5 - 1.3) | 0.18 | -0.7 |
| Z-Score d365 vs. before HSCT ^c^ | -2 (-2.3 - 0.1) | -0.5 (-1 - 0.24) | **0.002** | **-1.5** |
| Discolored or green stool, n (%) | 11 (91.7) | 25 (78.1) | 0.41 | 2.4 |
| Diarrhea during HSCT-hospitalization, n (%) | 13 (100) | 29 (90.6) | 0.55 | - |
| Diarrhea episodes, n ^c^ | 8 (1-13) | 3 (0-23) | **0.007** | **+5** |
| Parenteral nutrition duration [days] ^c^ | 98 (23-269) | 60 (20-365) | 0.20 | +14 |
| No or only little liquid food intake in week 2 after HSCT | 13 (100) | 24 (75) | 0.08 | 1.3 |
| **C - Immune reconstitution** | **s.pEPI**  **n=13** | **Ctrl**  **n=32** | **p-value ^a^** | **Effect size ^b^** |
| B cell count at d365 [/nl] ^c^ | 0.1 (0-1.0) | 0.6 (0-1.2) | 0.10 | -0.5 |
| Neutrophil count >500/µl [days after HSCT] ^c^ | 19 (12-60) | 19 (9-43) | 0.63 | 0 |
| Neutrophil count >1000/µl [d] ^c^ | 19 (12-60) | 22 (9-43) | 0.75 | -3.0 |
| Platelet count >50000/µl [days after HSCT] ^c^ | 38 (2-144) | 31 (1-259) | 0.90 | +7 |
| Lymphocyte count at d365 [/nl] ^c^  CD3+ count at d365 [/nl] ^c^  CD4+ count at d365 [/nl] ^c^  CD8+ count at d365 [/nl] ^c^ | 2.1 (0.3-3.6)  1.5 (0.1-3.0)  0.3 (0.1-0.6)  1.3 (0-3.2) | 1.9 (0.2-4.3)  1.3 (0.1-2.9)  0.7 (0.1-1.9)  0.6 (0.1-1.2) | 0.82  0.72  0.20  0.14 | +0.2  +0.2  -0.4  +0.7 |
| Total chimerism at d365 [%] ^c^  CD34 chimerism at d365 [%] ^c^ | 100 (98-100)  100 (99-100) | 100 (97-100)  100 (97-100) | 0.84  >0.99 | 0  0 |
| **D - Secondary complications** | **s.pEPI**  **n=13** | **Ctrl**  **n=32** | **p-value ^a^** | **Effect size ^b^** |
| Duration of hospitalization for HSCT [days] ^c^ | 117 (47-225) | 50 (29 236) | **0.0026** | **+67** |
| Cumulative re-hospitalization duration [days] ^c^ | 33 (6-78) | 12 (0-70) | **0.03** | +21 |
| Survived severe adverse events, n (%) ^h^ | 5 (38.5) | 3 (9.4) | **0.04** | **2.9** |
| Stem cell boost, n (%) | 2 (15.4) | 3 (9.4) | 0.62 | 1.5 |
| Donor lymphocyte infusions, n (%) | 0 (0) | 3 (9.4) | 0.55 | - |
| Viral infection (>2000 copies/ml in blood), n (%) | 12 (92.3) | 25 (78.1) | 0.40 | 2.6 |
| Number of viral infections with >2000 copies/ml per patient, n (%) |  |  |  |  |
| 1 | 1 (7.7) | 9 (28.1) | 0.24 | 0.3 |
| 2 | 1 (7.7) | 4 (12.5) | >0.99 | 0.7 |
| 3 | 4 (30.8) | 7 (21.9) | 0.70 | 1.4 |
| 4  5  ≥6 | 0 (0)  0 (0)  5 (38.5) | 2 (6.3)  1 (3.1)  2 (6.3) | >0.99  >0.99  **0.02** | -  -  **3.4** |
| EBV infection (>2000 copies/ml blood), n (%) | 4 (30.8) | 10 (31.3) | >0.99 | 1.0 |
| ADV infection (>2000 copies/ml blood), n (%) | 8 (61.5) | 8 (25.0) | **0.04** | **2.9** |
| ADV finding in feces n (%) | 9 (69.2) | 10 (31.3) | **0.04** | **3.1** |
| CMV infection (>2000 copies/ml blood), n (%) | 5 (38.5) | 8 (25.0) | 0.50 | 1.5 |
| HHV6 infection (>2000 copies/ml blood), n (%) | 4 (30.8) | 1 (3.5) | **0.03** | **3.3** |
| HHV6 finding in feces, n (%) | 4 (50.5) | 3 (23.1) | 0.35 | 2.0 |
| Cumulative occurrence of HHV6, EBV and/CMV | 10 (76.9) | 18 (56.3) | 0.3 | 1.4 |
| BKV infection (>2000 copies/ml blood), n (%) | 5 (38.5) | 9 (47.4) | 0.72 | 0.8 |
| BKV finding in urin, n (%) | 8 (61.5) | 9 (37.5) | 0.19 | 1.9 |
| Virus-specific T cells, n (%) | 2 (15.4) | 9 (28.1) | 0.47 | 0.6 |
| Neutropenia relapses <500/µl blood ^c, i^ | 1 (0 - 4) | 0 (0 - 4) | **0.04** | **1** |
| Initiations of intravenous antibiotic treatment ^c^ | 2 (1-5) | 2 (1-6) | >0.99 | 0 |
| Positive blood cultures, n (%) | 7 (53.9) | 13 (40.6) | 0.52 | 1.5 |
| Systemic antimycotic treatment in therapeutic dose, n (%) | 12 (100) | 24 (85.7) | 0.30 | - |
| Relevant acute GvHD, n (%) ^j^ | 5 (38.5) | 8 (25.0) | 0.47 | 1.5 |
| Relevant acute GvHD of the gut, n (%) ^j^ | 4 (30.8) | 2 (6.25) | **0.05** | **2.9** |
| Moderate - severe chronic GvHD of the gut, n (%) | 3 (23.1) | 1 (3.1) | 0.07 | 3.1 |
| Cumulative systemic steroid treatments ^c^ | 1 (0-5) | 1 (0-3) | 0.31 | 0 |
| Extra-corporal photopheresis (ECP), n (%) | 2 (15.4) | 3 (9.4) | 0.62 | 1.5 |
| Vedolizumab, basiliximab and/or infliximab, n (%) | 5 (38.5) | 11 (34.4) | >0.99 | 1.1 |
| Pancreatitis diagnosis, n (%) | 2 (15.4) | 1 (3.1) | 0.2 | 2.5 |
| Red blood cell concentrates per patient, n ^c^ | 30 (5-76) | 12 (3-61) | **0.025** | **18** |
| Red blood cell concentrates transfused (total), n | 448 | 475 |  |  |
| Platelet concentrates per patient, n ^c^ | 73 (3-295) | 12 (1-113) | **0.0013** | **+61** |
| Platelet concentrates transfused (total), n | 1158 | 736 |  |  |
|  |  |  |  |  |

^a^ To compare cohorts the Mann-Whitney U rank test was used for continuous data and the Fishers exact test for binary data. GraphPad PRISM 8 & 9 (GraphPad Software, San Diego, USA).

^b^ For comparisons of continuous data the *difference of median*, and for comparisons of binary data the *relative risk* are given as an indicator of the effect size.

^c^ Median (range).

^d^ Onset of s.pEPI was defined as the week of the first PE1 level <200µg/g within the s.pEPI defining time period

^e^ End point of s.pEPI was defined as last PE1 level <200µg/g of the defining time period with the following two consecutive measurements showing levels >200µg/g

^f^ As PERT was not part of the study design important information for assessing its influence is missing. Symptoms directly before and after start were not documented in a systematic manner. Information on compliance and correct use is missing for most patients. Hence, it was not feasible to analyze the effect of PERT in our study further and its effect on the study cohort has to be interpreted with caution. Initiation happened at a mean of 128 days of s.(p)EPI group allocation PE level abnormality onset.

^g^ In 3 cases insulin substitution coincided with glucocorticosteroid treatment for GvHD. The other 3 patients did not have an overt or documented reason for insulin-substitution dependent blood-glucose increase.

^h^ Oriented on common terminology criteria for adverse events (CTCAE) version 5.0 Grade 4: Complications that necessitated transfer to ICU, Catecholamines to prevent cardiac failure, dialysis, invasive ventilation.

^i^ New drop of neutrophil blood level below 1000/µl after previous regeneration of neutrophils (>1000/µl) for at least 7 days (independent of granulocyte colony stimulating factor treatment)

^j^ This includes every episode of acute GvHD for which patients received systemic treatment like steroids or extracorporeal photopheresis.

*s.p EPI: suspected prolonged Exocrine pancreatic insufficiency, HSCT: Hematopoietic stem cell transplantation, MUD: Matched unrelated donor, MSD: Matched sibling donor, MMRD: Mismatch related donor, MRD: Matched related donor, HSC:* Hematopoietic stem cell, *BM: Bone marrow, PBSC: Peripheral blood stem cells, BW: body weight. ADV: Adenovirus, CMV: Cytomegalovirus, HHV6: Human herpes virus 6, BKV: BK virus, PE: Pancreas elastase.*

# Supplementary Figures

## Figure S1

**Figure S1 - Overview**

Representation of Pancreatic Enzyme (PE) measurements, pancreas sonography findings, and occurrence of dropouts for each patient within the study cohort over time. Week “0” denotes the day of hematopoietic stem cell transplantation. Imaging displayed in week-2 was conducted anytime between 3 months prior to HSCT and week-2. *PE: Pancreas elastase*

## Figure S2

**Figure S2 - Exploratory testing of definitions of suspected (persistent) exocrine pancreas insufficiency shows that the used definition (III) exhibits the highest discriminatory power between s.(p)EPI and control patients regarding distribution of positive imaging.**

**(A)** Percentage of patients meeting the predefined s.EPI diagnosis criteria: I: ≥ 2x PE <200 [µg/g] within a period of min. 7 to max. 28 days with at least 7 days between two measurements. II: Same as I, with additional criteria including positive imaging within ±14 days of pathological PE finding, pathological PE level in a solid stool sample, or ≥ 2x PE-<100 µg/g during the defining time period. III: Same as I, with additional criteria as in II, excluding the ≥ 2x PE <100 µg/g requirement. The exact number of patients fulfilling each definition is provided below. **(B)** Percentage of patients fulfilling different s.EPI- or control group criteria with positive imaging findings during the defining time period ±14 days. **(C)** Percentage of patients meeting the predefined s.pEPI diagnosis criteria: Ia: Period of at least 8 weeks with PE <200 in the first and last week, with at least 75% of PE measurements < 200µg/g and at least 7 days between two measurements. Ib: Same as Ia, with 66% of PE measurements < 200µg/g. Ic: Same as Ia, with 50% of PE measurements < 200µg/g. II: Same as Ib, with additional criteria including positive imaging within ±14 days of pathological PE finding, pathological PE level in a solid stool sample, or ≥ 2x PE-<100 µg/g during the defining time period. III: Same as Ib, with additional criteria as in II, excluding the ≥ PE <100 µg/g requirement. The exact number of patients fulfilling each definition is provided below. **(D)** ±14Percentage of patients fulfilling different s.pEPI- or control group criteria with positive imaging findings during the defining time period days. **(E)** PE levels of all stool samples analyzed categorized by fecal consistency of the respective sample. **(F)** PE levels of all stool samples analyzed categorized by stool frequency on the day of analysis. **(G)** Correlation of fecal PE levels with food amount and composition across all patients and time points post-HSCT. **(H-K)** Mean serum insulin, serum C-peptide, mean daytime serum non-fasting glucose, and serum lipase levels per patient per time period. **(L,M)** Percentage of patients with mean daytime serum non-fasting glucose >150mg/dl and serum lipase <60U/l per time period. *d/w: day/week after HSCT, PE: Pancreas elastase, s.(p)EPI: suspected (prolonged) exocrine pancreas insufficiency, Ctrl: control group. HSCT: hematopoetic stem cell transplantation. PE: pancreatic elastase, d: day, w: week, n: sample size. Statistical analysis: (B, D, L, M): Fisher’s exact test. (E, F): Kruskal-Wallis test and Dunn post hoc test, (G) Spearman correlation.*

## Figure S3

**Figure S3 - Endpoint analysis after one year did not show significantly different outcomes in s.(p)EPI patients**

**(A)** Kaplan-Meyer curves depicting 1-year event-free survival for all patients as well as for the s.(p)EPI- and control groups during the first year after HSCT. **(B, D & C)** Inverse Kaplan-Meyer curve depicting the cumulative incidence of non-relapse mortality, transplant rejection and relapse for all patients as well as for the s.(p)EPI- and control groups during the first year after HSCT. (C) Inverse Kaplan-Meyer depicting the cumulative incidence of non-relapse mortality in s.pEPI- and control group in an extended period of up to 3 years post-HSCT. d/w: day/week after HSCT, s.*(p)EPI: suspected (prolonged) exocrine pancreas insufficiency, Ctrl: control group, n: number of subjects. Statistical analysis: (A-E: Kaplan-Meier statistics and log-rank test; n.s.: not significant.*

## Figure S4

**Figure S4 - Patients allocated to the s.(p)EPI groups show clinical findings typical for exocrine pancreas insufficiency**

**(A)** Mean PE level per patient per time period for s.(p)EPI- and control groups. **(B)** Comparison of PE levels of all solid and liquid stool samples between s.(p)EPI and control groups. **(C)** Comparison of mean rate of patients with mean PE levels <200µg/g stool per time period between s.(p)EPI- and control groups. **(D)** Comparison of mean rate of patients with loss of appetite per time period between s.(p)EPI- and control groups. **(E)** Comparison of mean food composition and amount per time period between s.(p)EPI- and control groups. **(F)** Inverse Kaplan-Meyer curve depicting the percentage of s.(p)EPI and respective control patients who started eating solid food (equals “4” in (E)) at different time points after HSCT. **(G)** Mean body weight Z-scores at different time points for s.(p)EPI and control groups. Z-scores were calculated from the weight percentile at the given time point as a surrogate marker for the age- and sex-dependent body weight. *HSCT: hematopoietic stem cell transplantation, d/w: day/week after HSCT, PE: Pancreas elastase, s.(p)EPI: suspected (prolonged) exocrine pancreas insufficiency, Ctrl: control group. Statistical analysis: (A, C, D, E, G): mixed-model testing, (B): Mann-Whitney test, (F): Gehan-Breslow-Wilcoxon test; Where not specified otherwise: number of subjects are n(s.EPI) = 32, n(s.EPI-Ctrl) = 39, n(s.pEPI) = 13, n(s.pEPI-Ctrl) =32; n.s.: not significant.*

s.EPI 32 32 32 32 30 25 23 14

Ctrl 39 39 39 39 30 29 25 22

s.pEPI 13 13 13 13 13 10 10 5

Ctrl 32 32 32 32 31 27 22 15

**before d0 w1+2 d30 d60 d100 d180 d365**

**Sample size per time point (n=)**

## Figure S5

**Figure S5 - Immune reconstitution and endocrine pancreas function**

**(A)** Comparison of CD3+, CD4+, CD8+ and B cell counts at various time points after HSCT for s.(p)EPI- and control groups. **(B-D, G)** Comparison of Serum insulin-, C-peptide- and mean daytime non-fasting glucose and lipase levels at various time points for s.(p)EPI- and control groups. **(E and H)** Mean percentage of patients with (E) daytime serum non-fasting glucose >150mg/dl or (H) serum lipase <60U/l per time period in s.(p)EPI- and control groups. **(F)** Correlation analysis of PE levels in feces (Y-axis) against non-fasting glucose (X-axis) before and after HSCT for s.(p)EPI and control groups. *HSCT: hematopoietic stem cell transplantation, d/w: day/week after HSCT, PE: Pancreas elastase, s.(p)EPI: suspected (prolonged) exocrine pancreas insufficiency, Ctrl: control group, n: sample size. Statistical analysis: (A-E, G, H): mixed-model testing, F: Spearman correlation testing; Where not specified otherwise: sample sizes are n(s.EPI) = 32, n(s.EPI-Ctrl) = 39, n(s.pEPI) = 13, n(s.pEPI-Ctrl) = 32; n.s.: not significant.*

s.EPI 32 32 32 32 30 25 23 14

Ctrl 39 39 39 39 30 29 25 22

s.pEPI 13 13 13 13 13 10 10 5

Ctrl 32 32 32 32 31 27 22 15

**before d0 w1+2 d30 d60 d100 d180 d365**

**Sample size per time point (n=)**

## Figure S6

**Figure S6 - PE correlates inversely with calprotectin after HSCT**

**(A)** Comparison of mean calprotectin levels in stool samples of s.(p)EPI patients against the respective control groups per time period. **(B)** Mean percentage of patients with stool calprotectin >50µg/g per time period in s.(p)EPI- and control groups. **(C)** Correlation analysis of calprotectin level in feces (Y-axis) against PE levels in feces (X-axis) before and after HSCT for s.(p)EPI- and control groups. **(D)** Comparison of co-occurrence of pathological PE and calprotectin findings between grade III/IV gut GvHD- and control group using a four-fields box data representation. **(E)** Overlap in the cumulative incidence of s.EPI with °III/IV GI GvHD and/or infection post-HSCT of all patients with sufficient PE measurements and pancreas imaging for s.EPI group allocation (n=71). **(F)** Comparison of occurrence rates of abnormal PE levels or positive pancreatic imaging findings between patients with °III/IV GI GvHD or HHV6 infection and control patients (patients without GvHD or HHV6 infection respectively). Analyses were conducted using the two general cohorts from secondary endpoint analysis (1) and (2), (Fig. 2): All patients with sufficient PE measurements for s.EPI group allocation ((1) s.EPI prerequisite, n=83) or patients with sufficient PE measurements for s.pEPI group allocation ((2) s.pEPI prerequisite, n=51).

s.EPI 32 32 32 32 30 25 23 14

Ctrl 39 39 39 39 30 29 25 22

s.pEPI 13 13 13 13 13 10 10 5

Ctrl 32 32 32 32 31 27 22 15

**before d0 w1+2 d30 d60 d100 d180 d365**

**Sample size per time point (n=)**

## Figure S7

**Figure S7 - Clinical parameters of patients with GI GvHD and ADV.**

**(A)** Mean PE level per patient per time period for the GI GvHD-, ADV- and respective control groups. **(B)** Comparison of initial HSCT hospitalization duration between GI GvHD-/ADV- and control groups. **(C)** Inverse Kaplan-Meyer curves depicting the rate of GI GvHD-/ADV- and control patients independent from parenteral nutrition over time. **(D)** Comparison of mean BMI at different time points for GI GvHD- and ADV-patients and controls. **(E)** Comparison of mean body weight Z-scores at different time points for GI GvHD- and ADV-patients and controls. **(F)** Mean food composition and amount per time period for GI GvHD-, ADV- and control groups. **(G)** Inverse Kaplan-Meyer curve depicting the percentage of GvHD-, ADV- and respective control patients who started eating solid food (equals “4” in (F)) at different time points post-HSCT. **(H)** Mean percentage of patients with loss of appetite per time period in GvHD-, ADV- and control groups. *HSCT: hematopoietic stem cell transplantation, d/w: days/weeks after HSCT, PE: Pancreas elastase, Ctrl: control group. GI GvHD: Gastrointestinal Graft-versus-host-disease, ADV: Adenovirus, n: sample size. Statistical analysis: (A, D, E, F, H): mixed model analysis, (B): Mann-Whitney test, (C, G): Gehan-Breslow-Wilcoxon test; Where not specified otherwise: sample sizes are n(GvHD) = 10, n(GvHD-Ctrl) = 73, n(ADV) = 33, n(ADV-Ctrl) =50; n.s.: not significant.*

GvHD 10 10 32 32 30 9 8 7

Ctrl 73 73 67 66 60 55 42 30

ADV 33 33 33 29 29 25 19 13

Ctrl 50 50 49 42 41 39 30 23

**before d0 w1+2 d30 d60 d100 d180 d365**

**Sample size per time point (n=)**

## Figure S8

**Figure S8 - Co-occurrence of ADV and other viral infections in s.EPI and control patients.**

**(A)** Comparison of occurrence of non-ADV viral infections (HHV6, EBV and/or CMV) in s.EPI- and control patients. **(B)** Comparison of time-independent co-occurrence of non-ADV viral infections (HHV6, EBV and/or CMV) with ADV between EPI- and control patients. **(C)** Overlap in the cumulative incidence of s.(p)EPI with ADV and/or other viral infections post-HSCT. Included are all patients with sufficient PE measurements and pancreas imaging for s.EPI group allocation (n=71). **(D)** Tabular overview of time-independent co-occurrence rates of ADV with other viruses (HHV6, EBV, and CMV) in the s.EPI group versus the control group. Effect sizes are reported as relative risk, and p-values were determined using Fisher’s exact test. **(E)** Tabular overview of co-occurrence rates of ADV with other viruses (HHV6, EBV, and CMV) before s.EPI onset compared to patients with ADV without subsequent s.EPI. Effect sizes are reported as relative risk, and p-values were determined using Fisher’s exact test. *ADV: Adenovirus, Ctrl: control group, d/w: days/weeks after HSCT, GvHD: Graft-versus-host-disease, HSCT: hematopoietic stem cell transplantation, PE: Pancreas elastase, n: sample size, s.(p)EPI: suspected (prolonged) exocrine pancreas insufficiency. Statistical analysis: (A, B, D, E): Fisher’s exact test.*
